# Color analysis of horticultural produces using hue spectra fingerprinting

**DOI:** 10.1016/j.mex.2021.101594

**Published:** 2021-11-30

**Authors:** Lien Le Phuong Nguyen, László Baranyai, Dávid Nagy, Pramod V. Mahajan, Viktória Zsom-Muha, Tamás Zsom

**Affiliations:** a Hungarian University of Agriculture and Life Sciences, Institute of Food Science and Technology, Budapest, Hungary; b Industrial University of Ho Chi Minh City, Institute of Biotechnology and Food Technology, Ho Chi Minh, Viet Nam; c Department of Horticultural Engineering, Leibniz Institute for Agricultural Engineering and Bioeconomy (ATB), Potsdam, Germany

**Keywords:** Fruit and vegetable color, Machine vision, Digital image processing, Postharvest technology

## Abstract

Color has great importance in agriculture due to its relationship with plant pigments and therefore, plant development and biochemical changes. Due to the trichromatic vision, instruments equipped with CCD or CMOS sensor represent color with the mixture of red, green and blue signals. These values are often transformed into HSL (hue, saturation, luminance) color space. Beyond average color of the visible surface area, histograms can represent color distribution. Interpretation of distribution can be challenging due to the information shared among histograms. Hue spectra fingerprinting offers color information suitable for analysis with common chemometric methods and easy to understand. Algorithm is presented with GNU Octave code.

• Hue spectra is a histogram of hue angle over the captured scene but summarizes saturation instead of number of pixels. There are peaks of important colors, while others of low saturation disappear. Neutral backgrounds such as white, black or gray, are removed without the need of segmentation.
• Color changes of fruits and vegetables are represented by displacement of color peaks. Since saturation is usually changing during ripening, storage and shelf life, peaks also change their shape by means of peak value and width.

## Graphical abstract

**Figure:**
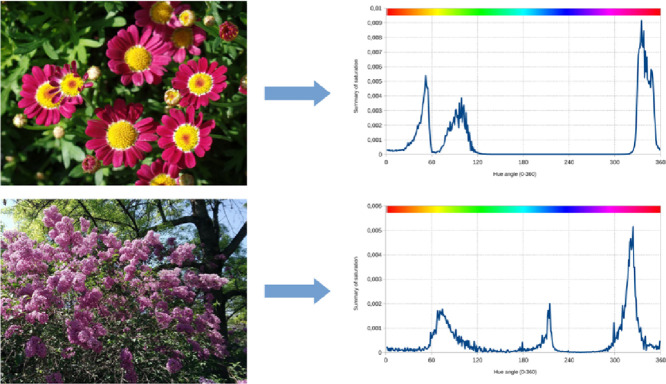

Specifications table

**Table:** 

Subject Area:	Agricultural and Biological Sciences
More specific subject area:	Biophysics, Machine vision, Digital image processing
Method name:	Hue spectra fingerprinting
Name and reference of original method:	Baranyai L., Szepes A. (2002) Analysis of fruit and vegetable surface color. *Machine Graphics & Vision*, 11(2/3): 351-361. Nguyen, L.P.L., Visy, A., Baranyai, L., Friedrich, L., Mahajan, P.V. (2020) Application of hue spectra fingerprinting during cold storage and shelf-life of packaged sweet cherry. *Journal of Food Measurement and Characterization*, 14: 2689-2702.
Resource availability:	Function was implemented in GNU Octave to extract hue spectra of images.

## Method details

Color is an important parameter of produces of agriculture. Since pigments of fruits and vegetables usually change during ripening, color is often used as primary indicator of quality. Plant pigments are classified into four major groups: chlorophylls, carotenoids, flavonoids, and betalains [1]. Chlorophylls capture light for photosynthesis and provide the energy required for plant development and growth. Chlorophylls make the green color of land plants and green algae. Carotenoids, flavonoids, and betalains are accessory pigments with complementary absorbance spectrum to chlorophyll. They are secondary metabolites of plants with wide range of functionality and structure. Carotenoids (carotenes and xanthophylls) provide orange, yellow, pink or red colors of produces, such as citrus fruits, sweet corn, banana, carrot and pepper. Flavonoids are responsible for purple, blue, yellow and red color of produces, such as blueberry, blackberry, eggplant and plum. This group includes anthocyanins and frequently investigated with the antioxidant capacity of fruits. Betalains provide red, violet, orange and yellow color of produces, such as dragon fruit, cactus pear and beet. Betalains (betacyanins and betaxanthins) differ from anthocyanins in the chemical structures and some properties, but share similarities to anthocyanins in the color spectra, biological functions, and other properties [1]. All mentioned pigments fit into the visible wavelength range of 400–700 nm. As a result, color and color change of fruits and vegetables can be measured by machine vision systems. Instruments usually report average color indices for the observed small area (local color information), but image processing can compute histograms of the whole surface and provide information on color pattern as global color information. Evaluation of such histograms can detect defects, monitor ripening of fruits and vegetables [2].

Acquired color images represent color with the mixture of red (R), green (G) and blue (B) signals. So called true color pictures use 24 bit/pixel color depth, scaling each color signal on byte values of 0 – 255 (where 255 = 2^8^-1). The basic RGB signals can be transformed into HSV (hue, saturation, value) color space. First, the 2-dimensional location of the color point is calculated according to Eq. (1).

(1) $$\begin{array}{c} x=R-\;\frac{1}{2}\left(G+B\right)\\ \;y=\frac{\sqrt{3}}{2}\left(G-B\right) \end{array}$$

The x and y coordinates can be used to compute HSV color parameters Eqs. (2), ((3)):

(2) $$H_{RGB}=\tan^{-1}\frac{y}{x}$$

(3) $$S_{RGB}=\;2\sqrt{x^{2}+y^{2}}$$

These hue and saturation values Eqs. (2), ((3)) differ from standard CIE 1976 (L*a*b*) system definition as they are computed from acquired intensity values instead of a* and b*. In HSV color space, value (V) is average of RGB signals, while in HSL color space the relative luminance (L) is calculated with weighted summary. This latter lightness parameter is not included in the computation of hue spectra. Although picture lightness is ignored, too dark images can negatively affect the resolution of hue spectra. Due to the division in Eq. 2, function atan2 is used in code to prevent division by zero. Hue angle values have to be corrected since atan2 results are in the range from -180° to +180°. The RGB and hue, saturation values of common colors are presented in Table 1.

**Table 1:** Red, green and blue intensity values with hue and saturation for common colors.

Name	Red	Green	Blue	Hue°	Saturation
Black	0	0	0	0	0
Gray 50%	127	127	127	0	0
White	255	255	255	0	0
Red	255	0	0	0	255
Green	0	255	0	120	255
Blue	0	0	255	240	255
Yellow	255	255	0	60	255
Magenta	255	0	255	300	255
Cyan	0	255	255	180	255

To ensure reproducibility and comparability of readings, camera adjustment should be standardized, such as gamma correction and white balance. Since hue angle is calculated from captured red, green, and blue intensity values, the correct white balance is essential. Illumination also plays an important role. Additionally to the requirement of neutral color, the frequency of power supply may affect image quality as well. In case some fluctuation is expected in illumination color, or pictures of different instruments are compared, picture colors need adjustment to a standard. Typically, a color calibration chart is used in the background for this purpose [3,4]. Besides a complete color calibration chart, constant background color can also play this role. Such constant background can be used to standardize pictures of the same machine vision installation [5].

**Fig. 1:**
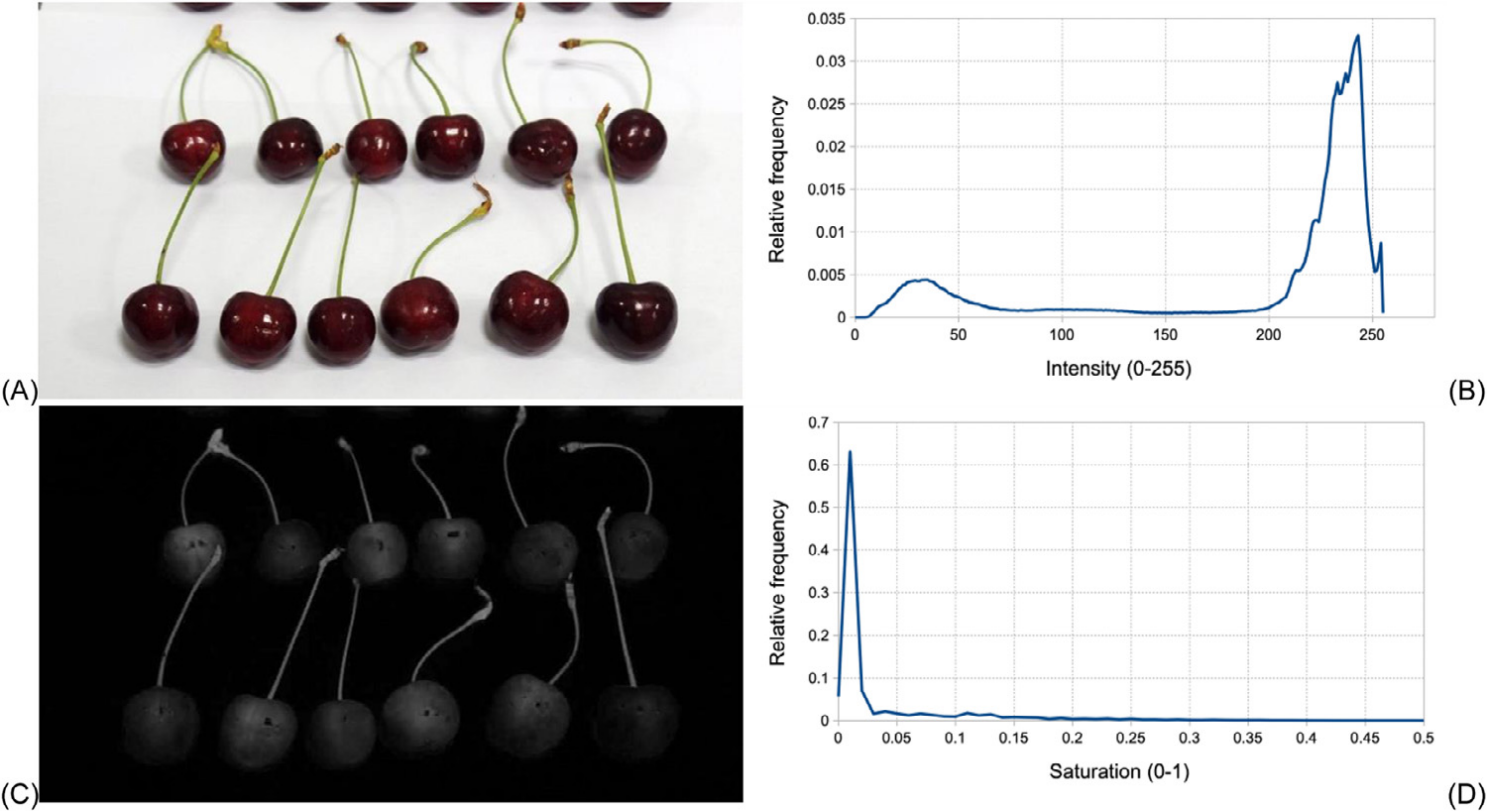
Example picture of fruit color characterization on white background (A), its intensity histogram (B), saturation image (C) and saturation histogram (D).

Fig. 1 shows an example picture of fruit color characterization with intensity and saturation histograms of the image. There is white background and fruit are darker with red (berry) and green (stem) color. The saturation histogram shows high frequency for low saturation values close to 0. That peak belongs to the background. Removing pixels of very low saturation results in segmentation of fruit. Saturation histogram does not have visible peaks above background peak, due to the very high amount of those pixels. The saturation image shows both fruit and stem on the picture (Fig. 1C). In case hue histogram is computed with summary of saturation values, background is automatically eliminated without the need of segmentation. Since white background still obtained small saturation values other than zero, it is recommended to adjust a threshold value during computation of hue spectra. According to authors’ experience so far, the value 0.05 (in the range of 0–1) is a suitable default threshold.

Table 2 shows the implementation of hue spectra computation using GNU Octave software. The code separates color layers of the image, RGB signals are stored in variables *pr, pg* and *pb*. Calculation follows the procedure of Eqs. (1)–(3). Saturation values are normalized into the range of 0 – 1. Hue angle values are rounded during transformation of double values to 16 bit unsigned integer. The summary values are divided by the total number of pixels. This normalization provides hue spectra comparable among images of different size.

**Table 2:** Implementation of hue spectra computation in GNU Octave.

function [RV]=huespectra(mpic,tv=0.05)
Layers = 0;
[Height,Width,Layers] = size(mpic);
# Check for color picture matrix
if Layers == 3
# get RGB color layers
pr = double(mpic(:,:,1));
pg = double(mpic(:,:,2));
pb = double(mpic(:,:,3));
# color point coordinates
dx = pr - pg/2 – pb/2;
dy = (pg – pb)*sqrt(3)/2;
# saturation, the distance from color space origin
spic = sqrt(dx.^2 + dy.^2)/255;
# hue, the color angle in degree
hpic = atan2(dy,dx)*180/pi;
hpic(hpic<0) += 360;
# select pixels above threshold
fc = uint16(hpic(spic>tv));
fc(fc==0) += 360;
fs = spic(spic>tv);
# collect spectra data
N = length(fc);
RV = zeros(1,360);
if N > 1
for i=1:N
RV(fc(i)) += fs(i);
endfor
RV /= N;
endif
else
printf('Hue Spectra Error: Non-color image matrix!\n');
RV = 0;
endif
endfunction

**Fig. 2:**
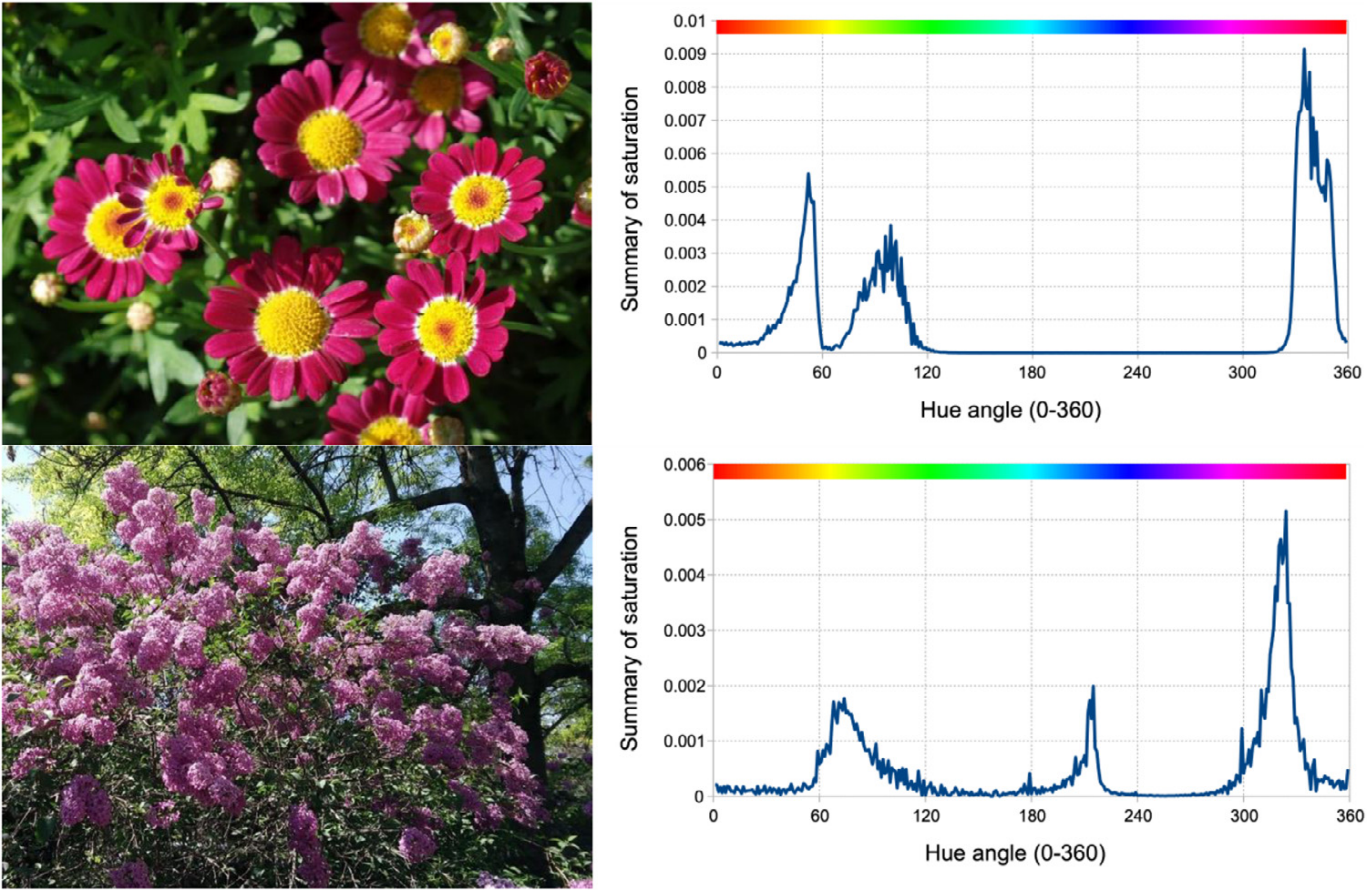
Example flower pictures (left) and their hue spectra (right).

Fig. 2 shows example pictures with their hue spectra. Pictures were taken in the botanic garden of Hungarian University of Agriculture and Life Sciences, Budapest. The upper color picture (Fig. 2) has yellow and red-purple flower over green background. Its hue spectrum consists three peaks for those colors. The lower color picture (Fig. 2) has pink color in front of green background and the blue sky is also visible. The lower hue spectrum has its largest peak near magenta color (Table 1).

Hue spectra can be processed using chemometric methods, such as partial least squares regression (PLSR). Similar mathematical methods are able to find relationship between color and other reference parameters, like soluble solids content (SSC), firmness, etc. There is a technique in near infrared spectroscopy (NIRS), which can be recommended to compress hue spectra shape information into single 2-dimensional point. Hue spectra is transformed to polar plot and the gravity point of the visible shape is used. This method is called Polar Qualification System (PQS) [6]. The gravity point (PQSx, PQSy) is computed based on triangular decomposition of shape (Eq. (4)), where V_l_ is the value of spectra at hue angle l, T is the total surface area of the polar figure, a is the angle between consecutive spectra values.

(4) $$\begin{array}{c} T=\frac{1}{2}\sum V_{\lambda i}V_{\lambda \left(i+1\right)}\sin\alpha \\ \text{PQS}x=\frac{1}{6T}\sum \;\left[V_{\lambda i}\cos\left(i\alpha \right)+V_{\lambda \left(i+1\right)}\cos\left(\left(i+1\right)\alpha \right)\right]\;V_{\lambda i}V_{\lambda \left(i+1\right)}\sin\alpha \\ \text{PQS}y=\frac{1}{6T}\sum \;\left[\;V_{\lambda i}\sin\left(i\alpha \right)+V_{\lambda \left(i+1\right)}\sin\left(\left(i+1\right)\alpha \right)\right]\;V_{\lambda i}V_{\lambda \left(i+1\right)}\sin\alpha \end{array}$$

Optimization of hue spectra analysis is possible with selection of the range of interesting colors. PQS might respond more sensitively to changes with optimized input.

## Method validation

**Fig. 3:**
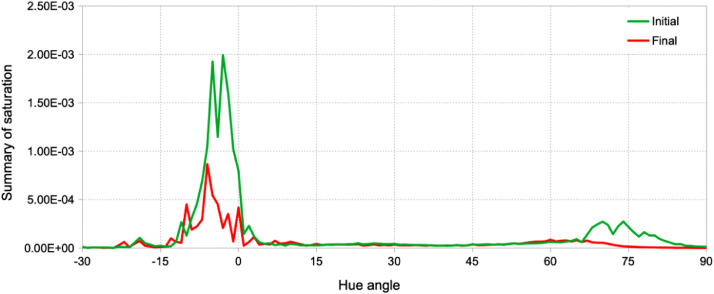
Average hue spectra of sweet cherry samples at the beginning (initial) and at the end of shelf life.

Sweet cherry (*Prunus avium* L. ‘Hudson’) color was monitored during 9 d cold storage at 10 ± 0.5°C and following 2 d shelf life at 20 ± 0.5°C [5]. Reference parameters of respiration, weight loss, firmness and total soluble solids content (TSS) were measured. Sweet cherry samples were placed on white background and this background was used as color reference. It was observed that hue spectra had peaks in the range of red and green colors, as expected. During the experiment, both red and green peaks were found to change value and green peak moved toward red (brown) color, as well. The average hue spectra of the cherry samples at the beginning and end of the experiment are presented on Fig. 3. The red and green regions were selected on the figure. PLSR models were made for prediction of reference parameters using hue spectra of color images. The best prediction was achieved for total soluble solids content with R^2^ = 0.972 and RMSE% = 0.706% for calibration and R^2^ = 0.683 and RMSE% = 2.546% for validation [5]. RMSE% (root mean squared error) parameter was calculated as relative error compared to measured value. The parameter TSS was followed by fruit firmness, respiration and weight loss, respectively. Besides TSS, only firmness prediction obtained low error with RMSE% = 1.596% for calibration and RMSE% = 7.578% for validation. According to the prior study, hue spectra was able to estimate two reference parameters and follow changes of sweet cherry during storage and shelf life.

Sweet cherry measurements were performed 5 times during the experiment, resulting 124 data points. The comparison of prediction efficiency of proposed hue spectra fingerprinting with multivariate regression (MVR) model utilizing common RGB average and standard deviation is presented in Table 3. The PLSR calibration models outperformed MVR ones, but validation achieved comparable results in terms of RMSE%.

**Table 3:** Comparison of sweet cherry prediction models PLSR of hue spectra and MVR of RGB data.

Quality parameter		Firmness		Total soluble solids (TSS)	
		Calibration	Validation	Calibration	Validation
R^2^	PLSR	0.979	0.672	0.972	0.683
	MVR	0.341	0.280	0.388	0.314
RMSE%	PLSR	1.572	8.626	0.688	3.387
	MVR	9.842	9.845	3.221	3.878

**Fig. 4:**
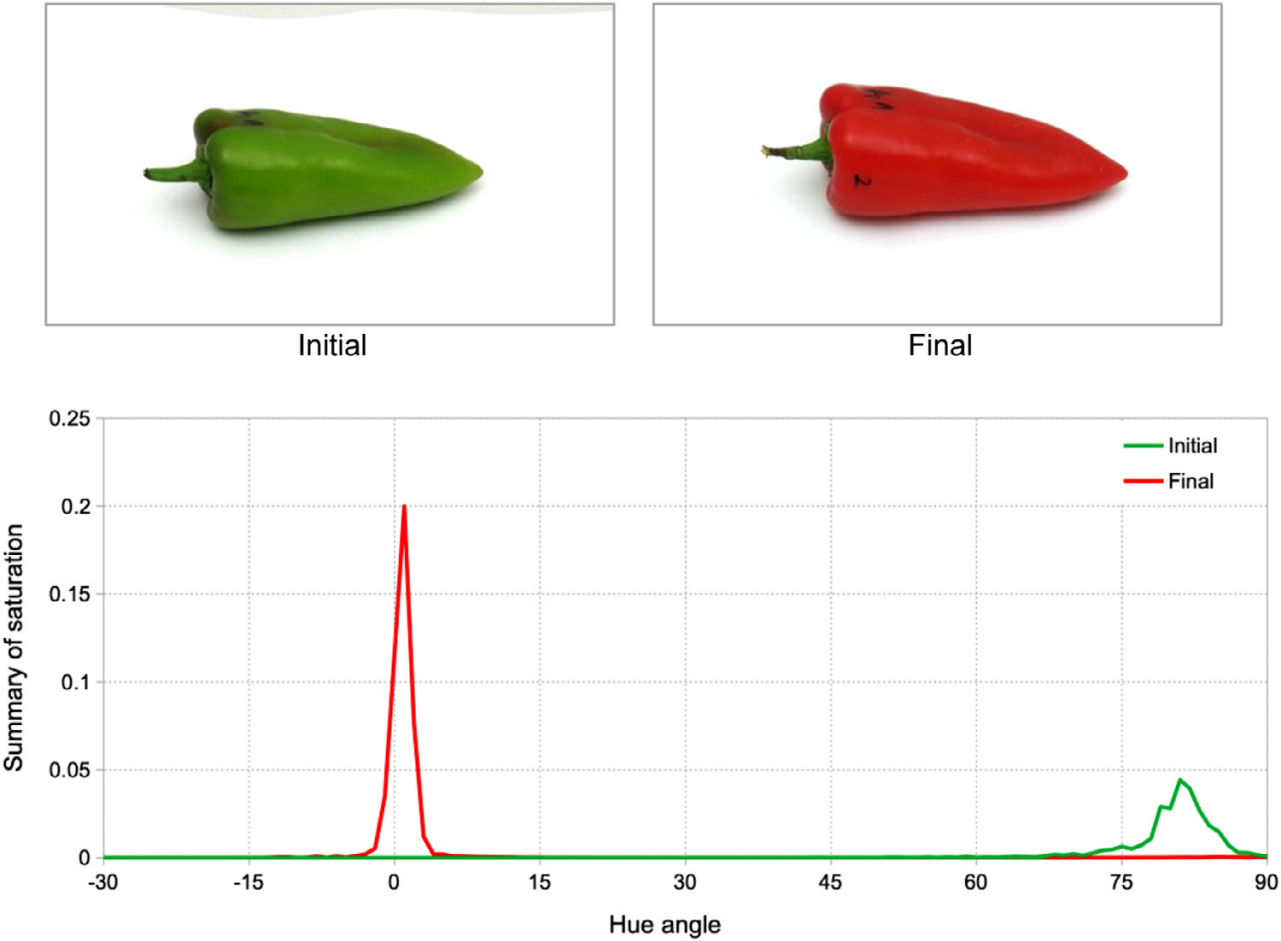
Kápia type sweet pepper samples during the experiment and their hue spectra.

In another study, Kápia type sweet pepper (*Capsicum annuum* L*. cv. ‘Kapitány’*) was monitored during 7 d cold storage followed by 7 d shelf life [7]. Fresh pieces were harvested for the experiment in semi-mature state called “turning” or “smoky green”. Initial samples were greener than red. This color was maintained until the end of cold storage, when color started changing toward red. At the end of shelf life, almost all pieces became red. Fig. 4 presents the same piece of pepper at the beginning and the end of the experiment with hue spectra of those pictures. The spectra (Fig. 4) were cut to highlight expected color range of red – green. Hue spectra of pepper had peaks at expected locations, representing smoky green and red colors.

PLSR models were made to predict reference parameters of acoustic firmness (Stiffness), mass loss, chlorophyll content (DA-index®) and chlorophyll fluorescence parameters (F_0_, F_M_, F_V_, F_V_/F_M_, F_M_/F_0_). These models were created for method validation, not published in prior study [7]. Due to the sudden change of color during the experiment, prediction models obtained low determination coefficients and high relative prediction error as R^2^ = 0.3283 and RMSE% = 14.97% for F_V_/F_M_, R^2^ = 0.3967 and RMSE% = 21.62% for F_M_/F_0_, R^2^ = 0.4986 and RMSE% = 28.54% for Stiffness. The chlorophyll content (DA-index®) obtained the highest R^2^ = 0.8675, which is promising but prediction error was too high. According to the observations of studies of sweet cherry [5] and sweet pepper [7], hue spectra behavior met expectations. Peak value, location and width were found to change during storage and shelf life of horticultural produces, following their color changes. Especially for cherry, PLSR calibration models achieved good efficiency in prediction of TSS and firmness based on hue spectra data.

## Declaration of Competing Interest

The authors declare that they have no known competing financial interests or personal relationships that could have appeared to influence the work reported in this paper.
